# Guidelines vs. generative AI in CKD patient education: the role of prompt engineering and expert blinded evaluation

**DOI:** 10.1186/s12882-026-04814-3

**Published:** 2026-02-20

**Authors:** Lutfullah Zahit Koc, Sevgi Gulsen Koc, Ayca Inci, Osman Cagin Buldukoglu, Gokhan Koker, Edgar V. Lerma

**Affiliations:** 1 Internal Medicine, Antalya Eğitim ve Araştırma Hastanesi, Antalya, Turkey; 2 Antalya Şehir Hastanesi, Antalya, Turkey; 3 Nephrology, Antalya Eğitim ve Araştırma Hastanesi, Varlik Mh. Kazim Karabekir Cd., Muratpaşa, Antalya, 07050 Turkey; 4 Gastroenterology, Antalya Eğitim ve Araştırma Hastanesi, Antalya, Turkey; 5 Nephrology, University of Illinois at Chicago, Chicago, USA

**Keywords:** Chronic kidney disease, Generative AI, Large language models, Patient education, Prompt engineering

## Abstract

This study aimed to evaluate the accuracy, content quality, and readability of patient education responses related to chronic kidney disease (CKD) generated by large language models (ChatGPT-4o mini and Gemini) compared to guideline group. Fifteen frequently asked CKD-related questions were selected using global Google Trends data and posed to both AI models and guideline-based sources. Responses were anonymized and evaluated by four independent nephrology professors using the CLEAR Tool, assessing completeness, appropriateness, evidence basis, and clarity. Both AI models significantly outperformed guideline responses across all CLEAR Tool domains (p < 0.001), with ChatGPT-4o mini achieving the highest median score (21.0 [IQR: 5.0] vs. Gemini: 17.0 [IQR: 5.0], Guideline: 13.0 [IQR: 2.0]). Initial readability analysis showed that guideline responses were easier to comprehend (Flesch-Kincaid Grade Level (FKGL): 9.40; Flesch Reading Ease (FRE): 52.01) than AI-generated content (ChatGPT FKGL: 11.34, FRE: 36.17; Gemini FKGL: 9.62, FRE: 46.36). However, when a structured prompt was applied, AI responses demonstrated significant improvements in readability, reducing the required literacy level to approximately the 7th-grade (ChatGPT FKGL: 7.87, FRE: 64.23; Gemini FKGL: 7.13, FRE: 61.45). These findings highlight the potential of prompt-guided AI models to generate accurate, accessible educational content for CKD. Prompt engineering emerges as a practical tool to enhance clarity and usability, particularly for populations with limited health literacy. Integration with frameworks like Retrieval-Augmented Generation may further improve reliability and safety in digital health communication.

**Supplementary Information:**

The online version contains supplementary material available at 10.1186/s12882-026-04814-3.

## Introduction

Chronic kidney disease is an increasing global public health issue, affecting approximately 700 million people worldwide. The prevalence of CKD has reached 9.1% globally, and the mortality rate associated with this disease across all age groups increased by 41.5% from 1990 to 2017 [1]. Known as the “silent epidemic,” this disease is often diagnosed at advanced stages due to its asymptomatic nature in early phases and low awareness levels [2]. Furthermore, low socioeconomic status and limited health literacy accelerate the progression of the disease, increasing the risk of progression to end-stage renal disease [3]. Enhancing health literacy plays a crucial role in slowing disease progression by enabling patients to gain knowledge about CKD. Creating awareness in the early stages and implementing appropriate treatment strategies not only improve quality of life and reduce mortality and morbidity rates but also decrease the need for high-cost treatments such as dialysis [3, 4].

In recent years, artificial intelligence (AI) technologies have introduced a new paradigm for enhancing health literacy and disease management. The rapid advancements in AI technologies hold significant potential to improve accessibility to healthcare services and democratize access to information [5]. Generative AI models, particularly large language models(LLMs) such as ChatGPT-4o mini (by OpenAI, San Francisco, CA) and Gemini (by Google, Mountain View, CA), have become easily accessible to a broad range of users. These models have the potential to facilitate patients’ access to health information, providing personalized education and guidance [6, 7]. However, further research is needed to evaluate the reliability and accuracy of these technologies.

Strategies have been developed to enhance the accuracy of AI models in the medical field. One of the most commonly used strategies is prompt engineering, which aims to ensure that AI systems generate accurate, relevant, and reliable outputs. Prompt engineering involves designing and optimizing input instructions to Guideline AI models toward producing specific outputs. An structured prompt ensures that the generated response contains accurate and user-appropriate information [8, 9]. For example: “I am a patient with chronic kidney disease, and I have some questions about my condition. I would like to ask you these questions as if you are a nephrologist. Could you answer them in a way that an elementary school graduate can easily understand?” By explicitly stating the user’s condition (patient with chronic kidney disease), assigning a role (as a nephrologist), and tailoring the response to the user’s educational level (elementary school graduate), the likelihood of receiving accurate and relevant answers is increased.

Previous studies have demonstrated the potential of AI-supported tools in diverse healthcare contexts, such as providing personalized dietary and exercise recommendations for obesity and delivering reliable, easy-to-understand information for conditions like hypothyroidism during pregnancy [6, 7]. Building on these findings, this study is the first to systematically blinded compare AI-generated responses with guideline-based answers in the context of CKD. By integrating prompt engineering techniques, we aimed to improve both the readability and content accuracy of AI-generated educational materials.

## Materials and methods

**Fig. 1:**
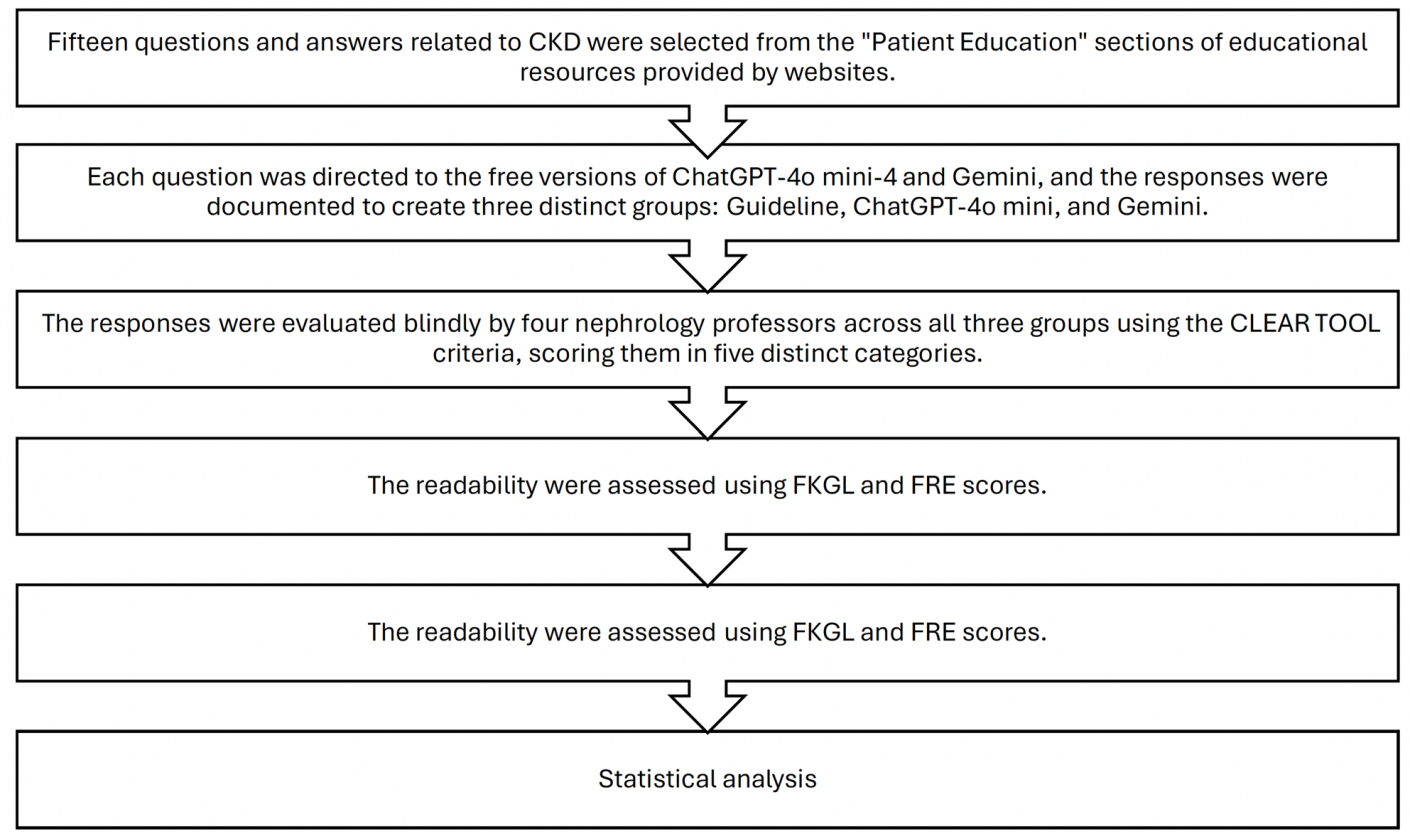
Study design and methodological workflow

This study was designed as a cross-sectional, comparative analysis to evaluate the accuracy, content quality, and readability of responses to selected patient questions about CKD. The overall methodology is summarized in Fig. 1.

Fifteen representative questions were selected based on two-year worldwide Google Trends data, focusing on globally searched patient queries related to CKD. This approach ensured thematic relevance and captured real-world informational needs observed in both clinical and digital settings. All questions are listed in Supplementary Table 1.

To establish a benchmark, guideline-based reference answers were compiled from the patient education sections of internationally recognized authoritative health organizations, specifically Kidney Care UK, the National Kidney Foundation, Mayo Clinic, and the National Health Service. The selection criteria focused on publicly accessible, evidence-based resources designed for patient guidance. Reference responses were curated by an expert nephrologist by directly extracting relevant information from these platforms (https://kidneycareuk.org/, https://www.kidney.org.uk/, https://www.mayoclinic.org/, https://www.kidney.org/, https://www.nhs.uk/) to preserve semantic integrity and ensure standard-of-care accuracy without alteration. These compiled responses formed the ‘’Guideline group’’. The same set of questions was then posed to both AI models: ChatGPT-4o mini and Gemini 1.5 Flash. To enhance reliability and reduce variability, each prompt was executed in two independent sessions. Upon qualitative review confirming that semantic consistency was maintained across sessions without significant deviations in medical accuracy or core recommendations, the dataset from the first session was retained for the primary analysis.

All responses—whether guideline-based or AI-generated—were anonymized and formatted in a standardized style. To enhance the precision of inter-rater reliability, the minimum criterion of three raters recommended in the literature [10] was exceeded, and four expert raters were included in the analysis process to minimize potential biases. Four independent nephrology professors, each with over 15 years of experience, evaluated the responses in a blinded manner using the CLEAR Tool scoring system. Evaluators were unaware of the origin of each response and scored them across five subdomains: completeness, lack of false information, evidence-based, appropriateness, and relevance. This blinded design was implemented to reduce potential bias and ensure objective assessment.

The CLEAR TOOL was developed based on a literature review of existing health information quality assessment frameworks, including DISCERN, Patient Education Materials Assessment Tool (PEMAT), and the Centers for Disease Control and Prevention Clear Communication Index. While not directly adapted from a single tool, CLEAR incorporates conceptual elements such as completeness, evidence, and clarity that are shared with these validated instruments. Furthermore, the original pilot testing demonstrated the tool’s psychometric reliability, reporting satisfactory internal consistency with Cronbach’s alpha values ranging from 0.669 to 0.981 and statistically significant inter-rater agreement (Cohen’s kappa) across diverse health topics [11]. Full scoring rubric for the CLEAR TOOL is provided in Supplementary Table 2.

In addition to content quality, the readability of each response was assessed using two validated linguistic metrics: the Flesch-Kincaid Grade Level (FKGL) and the Flesch Reading Ease (FRE) [12, 13]. These indices account for sentence length, word count, and syllable density. FKGL estimates the U.S. school grade level required to comprehend a text, while FRE assigns a score from 0 to 100, with higher scores indicating greater readability. This dual approach enabled a nuanced analysis of how accessible each response was to the average patient.

To evaluate the impact of prompt engineering, the same 15 questions were re-submitted to both AI models under two conditions: Unstructured prompt and with a structured prompt. The prompt was designed based on best practices from the Gemini for Google Workspace Prompting Guide and included four key elements: persona (“a nephrologist”), task (“answer questions about chronic kidney disease”), context (“from the perspective of a patient with CKD”), and format (“in simplified language understandable by an elementary school graduate”) [14]. The final structured prompt was: “I am a patient with chronic kidney disease, and I have some questions about my condition. Please answer them as if you are a nephrologist, but in a way that an elementary school graduate can easily understand.” To visually demonstrate these qualitative improvements, a side-by-side comparison of sample responses from the Guideline, Unstructured Prompt, and Structured Prompt groups is provided in Supplementary Table 3.

These unstructured and structured prompts was applied identically to both models. Each unstructured and structured prompt-based query was initiated in a separate private browser session to prevent memory retention and ensure independence between responses. All answers were collected under consistent conditions using the default temperature and token settings of the public versions of ChatGPT-4o mini and Gemini, as of March 2025.

### Statistical analysis

Descriptive statistics were calculated. Data normality was assessed using Kolmogorov–Smirnov and Shapiro–Wilk tests. For data following a normal distribution, descriptive statistics were expressed as mean and standard deviation, and group comparisons were assessed using one-way analysis of variance (ANOVA), with Tukey’s test applied for post hoc analysis. For data not following a normal distribution, descriptive statistics were presented as median values and interquartile ranges, and differences between groups were evaluated with the Kruskal-Wallis test. Post hoc analyses were conducted using the Mann-Whitney U test with Bonferroni correction. The evaluations of the professors regarding the responses were analyzed based on the total scores assigned for each question, using Intraclass Correlation to assess agreement.

## Results

The Intraclass Correlation Coefficient (ICC) for the total scores was calculated to assess agreement among evaluators. The ICC value was 0.55, indicating moderate agreement among the evaluators.

The groups were evaluated by comparing the ‘total score,’ derived from the sum of the CLEAR TOOL components, as well as each component individually. The median and interquartile range (IQR) of ‘total scores’ for each group were as follows: Guideline: Median = 13.0 (IQR = 2.0), ChatGPT-4o mini: Median = 21.0 (IQR = 5.0), Gemini: Median = 17.0 (IQR = 5.0).

**Fig. 2:**
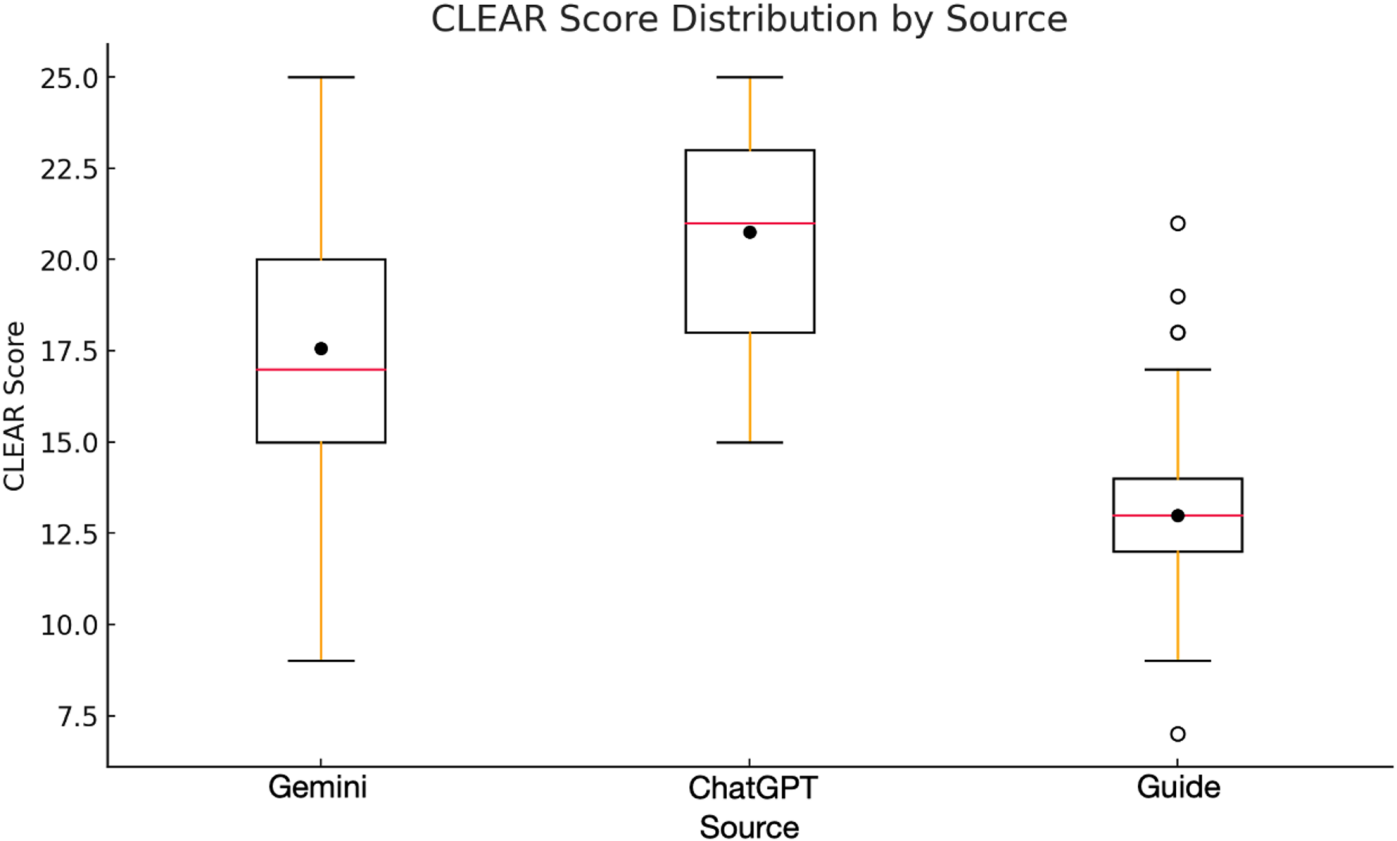
Box plot of total CLEAR scores for guideline, ChatGPT-4o mini, and Gemini responses

The Kruskal-Wallis test revealed a statistically significant difference in total scores between the three groups (H = 103.49, *p* < 0.001) (Fig. 2). Pairwise comparisons of the total scores using the Mann-Whitney U test with Bonferroni correction showed the following: Guideline vs. ChatGPT-4o mini: Median = 13.0 (IQR = 2.0) vs. 21.0 (IQR = 5.0), *p* < 0.001, Guideline vs. Gemini: Median = 13.0 (IQR = 2.0) vs. 17.0 (IQR = 5.0), *p* < 0.001, ChatGPT-4o mini vs. Gemini: Median = 21.0 (IQR = 5.0) vs. 17.0 (IQR = 5.0), *p* < 0.001.

When comparing the CLEAR TOOL components across the three groups, statistically significant differences were observed for all components. Post hoc analyses with Bonferroni correction (*p* = 0.05/3) revealed that both AI models (ChatGPT-4o mini and Gemini) differed significantly from the guideline group across all domains. However, differences between ChatGPT-4o mini and Gemini were not statistically significant in all components. (Table 1).

**Table 1:** Comparison of CLEAR Tool component scores across Guideline, ChatGPT-4o mini, and Gemini groups

CLEAR TOOL Component	Guideline Median (IQR)	ChatGPT-4o mini Median (IQR)	Gemini Median (IQR)	*p* value
Completeness	2 (IQR: 1)	5 (IQR: 1)	4 (IQR: 1)	*p*_*1*_ < 0.001*
				*p*_*2*_ < 0.001*
				*p*_*3*_ < 0.001*
Lack of false knowledge	3 (IQR: 1)	4 (IQR: 1)	4 (IQR: 1)	*p*_*1*_ < 0.001*
				*p*_*2*_ >< 0.001*
				*p*_*3*_ *=0,613*
Evidence based	3 (IQR: 1)	4 (IQR: 1)	3 (IQR: 1)	*p*_*1*_ < 0.001*
				*p*_*2*_ < 0.001*
				*p*_*3*_ < 0.001*
Appropriateness	3 (IQR: 1)	4 (IQR: 1.25)	3 (IQR: 1)	p_1_< 0.001*
				*p*_*2*_ *=0,503*
				*p*_*3*_ *=0,455*
Relevance	3 (IQR: 1)	4 (IQR: 2)	3 (IQR: 1)	*p*_*1*_ < 0.001*
				*p*_*2*_ *=0,293*
				*p*_*3*_ *=0,822*

Note: p1: Guideline-ChatGPT-4o mini, p2: Guideline-Gemini, p3: ChatGPT-4o mini-Gemini. Bolded comparisons indicate statistically significant differences after Bonferroni correction

## Readability

Readability levels were evaluated in two ways. First, questions were directly posed to AI tools (Unstructured Prompt), and the FKGL and FRE scores of their responses, along with Guideline answers, were calculated (Table 2).

For the Unstructured Prompt group, the Flesch–Kincaid Grade Level (FKGL) and Flesch Reading Ease (FRE) values for the Guideline, ChatGPT-4o mini, and Gemini groups were calculated as follows: Guideline: FKGL mean = 9.40 ± 2.29 (SD), FRE mean = 52.01 ± 14.85 (SD), ChatGPT-4o mini: FKGL mean = 11.34 ± 1.79 (SD), FRE mean = 36.17 ± 10.85 (SD), Gemini: FKGL mean = 9.62 ± 1.55 (SD), FRE mean = 46.36 ± 10.69 (SD).

Statistical analyses revealed a significant difference in FKGL between ChatGPT-4o mini and Guideline (*p* < 0.001), indicating that ChatGPT-4o mini’s responses required a higher educational level. Additionally, a significant difference was found between ChatGPT-4o mini and Gemini in FKGL scores (*p* < 0.001).

For FRE scores, significant differences were observed between ChatGPT-4o mini and Guideline (*p* < 0.001) as well as between ChatGPT-4o mini and Gemini (*p* < 0.001). ChatGPT-4o mini’s responses exhibited significantly lower readability scores, indicating greater difficulty in comprehension.

**Fig. 3:**
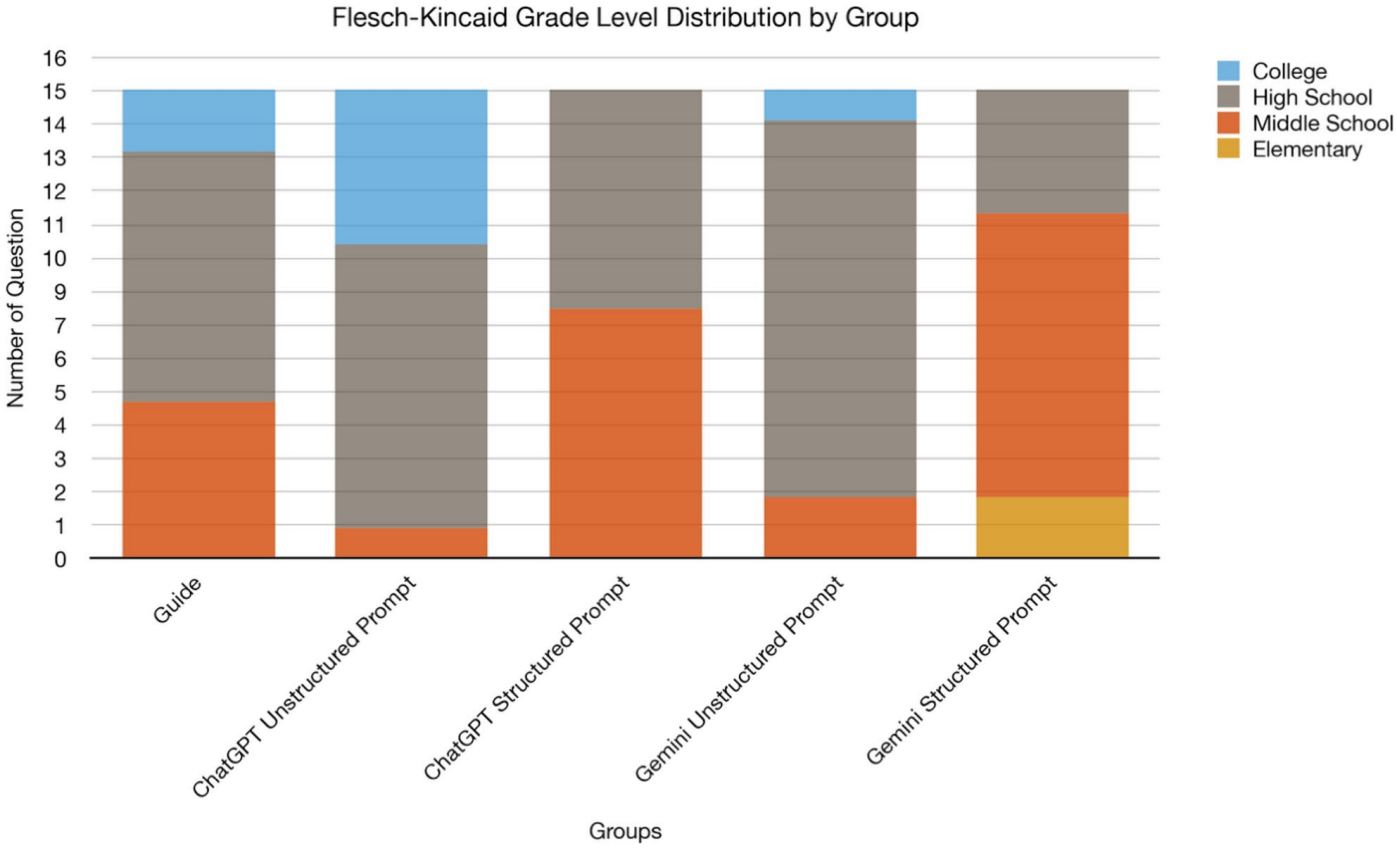
FKGL scores for guideline, ChatGPT-4o mini, and gemini responses unstructured and structured prompt

Guideline group texts were generally at a high school level (9th grade) and moderately easy to read. In contrast, ChatGPT-4o mini’s responses were at the level of a high school senior and posed greater comprehension challenges. Gemini’s texts were positioned between Guideline and ChatGPT-4o mini, requiring an educational level equivalent to the beginning of high school (Fig. 3).

When a structured prompt was provided, these readability metrics for the ChatGPT-4o mini and Gemini groups were calculated as follows (Table 2): ChatGPT-4o mini: FKGL mean = 7.87 ± 1.13 (SD), FRE mean = 64.23 ± 6.16 (SD), Gemini: FKGL mean = 7.13 ± 1.78 (SD), FRE mean = 61.45 ± 12.40 (SD).

Statistical analyses revealed a significant difference in both FKGL and FRE scores between the Structured Prompt and Unstructured Prompt groups for both ChatGPT-4o mini and Gemini (*p* < 0.001). Providing a structured prompt made the responses of both models significantly easier to read and reduced the required educational level to below high school (7th grade level).

With the use of a structured prompt, ChatGPT-4o mini’s responses became closer to the readability and comprehensibility levels of the Guideline group in terms of ease of understanding and educational level. Compared to unstructured prompt, the use of a structured prompt significantly improved the readability of responses and reduced their complexity for both ChatGPT-4o mini and Gemini (Fig. 3).

Statistical analyses confirmed these improvements: the FKGL and FRE scores of both models showed significant differences between structured prompt and unstructured prompt conditions (*p* < 0.001), based on Mann-Whitney U tests comparing paired responses across all 15 questions. These findings indicate that prompt engineering is an effective strategy to reduce linguistic complexity and enhance patient accessibility in AI-generated health content.

**Table 2:** Readability scores (mean ± SD) of FKGL and FRE for guideline, ChatGPT-4o mini, and Gemini responses, unstructured and structured prompt

Group	Unstructured Prompt		Structured Prompt		*p*-value
	FKGL Mean ± SD	FRE Mean ± SD	FKGL Mean ± SD	FRE Mean ± SD	
Guideline	9.40 ± 2.29	52.01 ± 14.85			
ChatGPT-4o mini	11.34 ± 1.79	36.17 ± 10.85	7.87 ± 1.13	64.23 ± 6.16	< 0.001*
Gemini	9.62 ± 1.55	46.36 ± 10.69	7.13 ± 1.78	61.45 ± 12.40	< 0.001*

Note: FKGL: Flesch-Kincaid Grade Level; FRE: Flesch Reading Ease; SD: Standard Deviation

## Discussion

This study is among the first known blinded comparative evaluations of AI-generated versus guideline-based responses in the context of CKD. It incorporates several methodological strengths that distinguish it from previous research. First, the evaluation was conducted by four independent nephrologists, a higher number of expert raters than typically employed in similar studies and literature, thereby improving the reliability and robustness of the results. Second, all responses—whether generated by AI or sourced from clinical guidelines—were evaluated under blinded conditions, minimizing potential bias. Unlike prior studies that often assess AI outputs against fixed references, this study applied a fully anonymized and independent evaluation protocol. Third, this study provides a holistic evaluation by integrating the assessment of content accuracy and quality with a comparative analysis of prompt engineering’s impact on readability and comprehensibility. Collectively, these contributions enhance the scientific rigor of the study and underscore its relevance for advancing AI-supported patient education in nephrology.

The results of this study align with existing literature. For instance, Zhang et al. demonstrated that ChatGPT-4o mini achieved 88% accuracy in providing accurate and relevant information on total knee replacement, underscoring its effectiveness in patient education [15]. Onder et al. found ChatGPT-4o mini-4’s responses reliable in hypothyroidism management, but readability analyses revealed a requirement for university-level education [7]. Similarly, Wang et al. evaluated ChatGPT-4o mini-4 and 4o-mini for clinical support in lumbar disc herniation and reported accuracy and completeness scores exceeding 75%; however, the responses were deemed “very difficult to read” [16].

Acharya et al. assessed 15 lifestyle and 20 dietary questions from Kidney Disease: Improving Global Outcomes (KDIGO) and Kidney Disease Outcomes Quality Initiative (KDOQI) guidelines answered by ChatGPT-4o mini-3.5, ChatGPT-4o mini-4, Gemini AI, and Bing AI. Responses were evaluated by nephrologists for accuracy. While the answers were generally accurate, misleading statements and irrelevant references were noted, particularly in ChatGPT-4o mini-3.5, ChatGPT-4o mini-4, and Gemini. All models delivered responses at a high school readability level, highlighting potential accessibility limitations for patients with low health literacy [17]. The tendency to generate inaccurate, out-of-context responses and provide incorrect references has been documented in other studies as well [9, 18].

In our study, the readability and comprehensibility of text generated by AI models significantly improved following structured prompt responses compared to unstructured prompt outputs. The reduction of FKGL to the 7th-grade level highlights the potential of this technology to effectively provide information to a broader patient population. By making responses more understandable, prompt engineering can facilitate access to information for individuals with low health literacy. This approach may serve as an effective tool for designing personalized patient education materials, thereby enhancing health literacy. Within this context, our study takes a step toward assessing the reliability of responses generated by LLMs such as ChatGPT-4o mini and Gemini in the healthcare domain. Beyond evaluating the accuracy and trustworthiness of these systems, the application of prompt engineering techniques has been found to enhance the readability and comprehensibility of AI-generated outputs. This contributes to producing more reliable and accessible content to counter misinformation. Notably, prompt engineering can serve as a valuable tool in improving AI-generated responses, making them more accurate and user-friendly, thereby reducing the spread of misinformation in healthcare.

With the widespread use of social media, the speed and scale at which misinformation spreads have greatly increased. A significant portion of fake news is crafted to be engaging and emotionally impactful, leading to higher interaction rates [19]. This, in turn, facilitates the increased use and rapid dissemination of false information across social media platforms. Such developments pose serious risks, especially in the healthcare sector. AI-generated content often relies on statistical probabilities, which may lead to misleading or out-of-context information lacking proper source validation [20]. While artificial intelligence can accelerate the spread of misinformation, it also holds significant potential for detecting and mitigating false information [21]. Various strategies have been implemented to combat misinformation and disinformation. Primarily, AI-powered misinformation detection systems on social media platforms use natural language processing and machine learning techniques to identify inaccurate content [22, 23]. However, research suggests that these systems should not only detect falsehoods but also encourage users to critically evaluate the credibility of the information they encounter [24]. In this regard, enhancing health literacy, raising public awareness, and fostering critical thinking through targeted educational initiatives are crucial. Additionally, it is essential for healthcare professionals and institutions to actively use social media to disseminate accurate information and counteract misinformation [19, 21]. On the other hand, relying on LLMs that utilize vast and unregulated datasets increases the risk of misinformation and hallucinations. To mitigate this, defining the operational boundaries of such models and promoting the development of smaller, domain-specific AI tools under clinical supervision may offer a safer alternative [25]. Models trained on validated medical corpora are more likely to produce clinically appropriate responses. Retrieval-Augmented Generation (RAG) and prompt engineering have been shown to reduce common accuracy issues and incorrect outputs in LLMs [8, 9, 26, 27].

RAG offers a promising approach to mitigate misinformation by grounding AI-generated responses in verified academic sources, such as KDIGO guidelines. By enabling real-time cross-validation with trusted references, RAG systems can enhance the factual accuracy and clinical reliability of AI outputs [25]. Integrating such targeted models into patient education—under the supervision of healthcare professionals and within regulated frameworks—could significantly reduce the dissemination of inaccurate information. This study provides a foundational step toward the application of RAG-based systems in CKD education and highlights their potential to strengthen the credibility and safety of AI-assisted health communication.

Despite its strengths, this study has several limitations. First, the analysis was based on only 15 questions, which may limit the generalizability of the findings. However, these questions were carefully selected from globally relevant search trends and validated sources to ensure maximum thematic representativeness. Second, the ICC was calculated at 0.55, which falls within the range of ‘moderate reliability’ (0.50–0.75) according to established guidelines [10]. A notable limitation regarding the standardization of the evaluation process must be acknowledged. Although the CLEAR tool is a structured assessment instrument, formal standardized rater training sessions or pilot coding exercises were not conducted prior to the main evaluation. However, unlike similar studies that typically employ only two raters to easily achieve higher agreement, we deliberately engaged a panel of four independent nephrologists. While achieving statistical consensus is inherently more challenging with a larger panel, this approach minimizes individual rater bias and reflects a more realistic diversity of clinical opinion. This highlights the need for more structured and consensus-driven approaches when assessing such outputs. In future studies, methods such as the Delphi technique, which involve multiple rounds of feedback to achieve expert consensus, may be beneficial—especially for evaluating subjective components like ‘appropriateness’ and ‘completeness.’ Employing this approach could help clarify evaluation criteria and reduce inter-rater variability, thereby enhancing methodological reliability. The AI models evaluated were free, publicly accessible versions, which may not fully reflect the capabilities of their enterprise-grade counterparts. Another limitation of this study is the absence of direct patient evaluation. While the ultimate goal is patient education, our study design prioritized expert validation in this initial phase to ensure clinical safety and accuracy as a necessary prerequisite, laying the groundwork for future patient-centered evaluations. Future studies should expand on these findings by incorporating more diverse and representative question sets, involving broader patient populations, and evaluating newer and clinically fine-tuned AI models.

While our study demonstrates that Structured Prompt Engineering effectively bridges the readability gap, reliance on general-purpose LLMs still inherently carries risks of hallucination. Therefore, we propose that the next evolutionary step in patient education is not merely better prompting, but the integration of Retrieval-Augmented Generation (RAG) systems. Although not implemented in the current study, RAG architectures can enhance safety by restricting AI responses to medical guidelines validated by healthcare professionals. Thus, the accessibility provided by AI can fill the critical gap in the improvement of health literacy.

## Conclusion

This study demonstrates that LLMs can outperform traditional Guideline-based content in both readability and overall informational quality when answering patient-centered questions about CKD. Among the evaluated tools, ChatGPT-4o mini consistently received higher scores across multiple domains, including completeness and appropriateness.

In addition, prompt engineering significantly enhanced the readability of AI-generated responses by lowering linguistic complexity and making content more accessible. The reduction in FKGL by over 3 grade levels underscores prompt engineering’s potential as a low-cost intervention to enhance health accessibility.

These findings highlight the growing capacity of well-designed AI tools to support patient education, particularly for populations with limited health literacy. Future studies should validate these results across broader clinical contexts and explore the safe, regulated integration of AI systems into routine health communication.

## Supplementary Information

Below is the link to the electronic supplementary material.

Supplementary Material 1

## Data Availability

The datasets used and/or analysed during the current study are available from the corresponding author on reasonable request.

## Abbreviations

AI: Artificial Intelligence
ANOVA: Analysis of Variance
CKD: Chronic Kidney Disease
FKGL: Flesch-Kincaid Grade Level
FRE: Flesch Reading Ease
ICC: Intraclass Correlation Coefficient
IQR: Interquartile Range
KDIGO: Kidney Disease: Improving Global Outcomes
KDOQI: Kidney Disease Outcomes Quality Initiative
LLMs: Large Language Models
PEMAT: Patient Education Materials Assessment Tool
RAG: Retrieval-Augmented Generation
SD: Standard Deviation
