# Supplementary material for: Guidelines vs. generative AI in CKD patient education: the role of prompt engineering and expert blinded evaluation
Source: BMC Nephrol. 2026 Feb 20;27:189. doi: 10.1186/s12882-026-04814-3 (PMC13032336; doi:10.1186/s12882-026-04814-3)
Supplement: Supplementary file 1 — Supplementary Material 1 [file 12882_2026_4814_MOESM1_ESM.docx]

| Selected Questions |
| --- |
| 1. Is chronic kidney disease rare condition? 2. What does it mean chronic kidney disease? 3. Is the dialysis only treatment for kidney disease? 4. Is chronic kidney disease genetic? 5. What are the symptoms of advanced chronic kidney disease? 6. Is drinking a lot of water good for the kidneys, especially in chronic kidney disease? 7. Could my back pain be due to my kidneys? 8. If I am at risk for kidney disease, is there nothing you can do about it? 9. Is chronic kidney disease curable? 10. What are the risk factors of chronic kidney disease? 11. What are the causes of chronic kidney disease? 12. As a chronic kidney disease patient, how should I eat? 13. What tests and procedures diagnose chronic kidney disease? 14. Is potassium harmful for the bodies? 15. Can I do excercise as a chronic kidney patient? |

**Supplementary Table 1: Selected questions.**

# **Supplementary Table 2: CLEAR TOOL Scoring Rubric**

Each response was evaluated across five domains: Completeness, Lack of False Information, Evidence Support, Appropriateness, and Relevance. Each criterion was rated on a 1 to 5 Likert scale. The rubric below was used to guide evaluators in assigning scores consistently.

| **Criterion** | **Score Range (1–5)** | **Scoring Guidelines** |
| --- | --- | --- |
| **Completeness** | 1 = Severely incomplete 5 = Fully comprehensive | Does the response address all major components of the question, providing sufficient detail? |
| **Lack of False Information** | 1 = Contains clear misinformation 5 = No false or misleading content | Is the response factually accurate and free from medical errors or misleading statements? |
| **Evidence Support** | 1 = No evidence or unsubstantiated claims 5 = Cites or reflects guideline-based evidence | Is the content grounded in current clinical guidelines or scientific consensus, even if not explicitly cited? |
| **Appropriateness** | 1 = Inappropriate tone or language 5 = Clinically appropriate and respectful | Is the tone professional, and is the information suitable for patient education without inducing fear or confusion? |
| **Relevance** | 1 = Largely irrelevant 5 = Fully aligned with the question asked | Does the response stay on-topic and directly address the patient's question without unnecessary digressions? |

**Supplementary Table 3:** Representative comparison of responses from Guideline, Unstructured AI, and Structured AI groups for a selected question. This comparison demonstrates the qualitative impact of prompt engineering on readability, tone, and clarity.

| **Group** | **Sample Response** |
| --- | --- |
| **Question** | What does it mean chronic kidney disease? |
| **Guide** | Chronic kidney disease (CKD) means your kidneys are damaged and can’t filter blood the way they should. The disease is called “chronic” because the damage to your kidneys happens slowly over a long period of time. This damage can cause wastes to build up in your body. CKD can also cause other health problems |
| **Chat GPT 4-o mini Unstructured Prompt** | Chronic kidney disease (CKD) is a long-term condition in which the kidneys gradually lose  their ability to function properly. The kidneys are vital organs responsible for filtering waste  products, excess fluids, and toxins from the blood, which are then excreted in the urine. They  also play a key role in regulating blood pressure, electrolyte balance, and red blood cell  production.  In CKD, the loss of kidney function occurs over months or years, and it is usually irreversible.  The condition is typically classified into five stages based on the glomerular filtration rate  (GFR), which measures how well the kidneys are filtering blood:  1. Stage 1: Kidney damage with normal or high GFR (≥90 mL/min/1.73 m2)  2. Stage 2: Mild decrease in GFR (60-89 mL/min/1.73 m2)  3. Stage 3: Moderate decrease in GFR (30-59 mL/min/1.73 m2)  Stage 3a: GFR 45-59 mL/min/1.73 m2  Stage 3b: GFR 30-44 mL/min/1.73 m2  4. Stage 4: Severe decrease in GFR (15-29 mL/min/1.73 m2)  5. Stage 5: Kidney failure (GFR <15 mL/min/1.73 m2), also known as end-stage renal  disease (ESRD), where dialysis or a kidney transplant may be needed.  CKD can result from various causes, including diabetes, hypertension, glomerulonephritis,  and polycystic kidney disease. Symptoms may not appear until the disease is advanced, which  is why early detection through screening and monitoring of risk factors is important.  Managing CKD involves addressing the underlying cause, slowing the progression of the  disease, and preventing complications. |
| **Chat GPT 4-o mini Structured Prompt** | Chronic kidney disease means your kidneys are damaged and have been working less well for a long time. The kidneys normally clean waste and extra water from your blood, but with this disease they cannot do that job as well. The word “chronic” means it is long-lasting and usually does not go away. Even though the damage cannot usually be fixed, treatments, medicines, and healthy habits can help slow it down and protect your kidneys. |
| **Gemini Unstructured Prompt** | Chronic kidney disease (CKD) is a condition where your kidneys gradually lose their ability to filter waste products and excess fluid from your blood. This damage happens slowly over months or years.  Think of your kidneys as filters in your car. If they become clogged or damaged, they can't effectively clean the impurities from your system. Similarly, when your kidneys are damaged, harmful substances can build up in your body, leading to various health problems. |
| **Gemini Structured Prompt** | Chronic Kidney Disease means your kidneys' tiny "cleaning filters" have been damaged or working more slowly than usual for at least three months. Instead of being a temporary illness that goes away like a cold, it is a long-term condition where the filters don't clean the "trash" out of your blood as perfectly as they once did. Because these filters usually don't fix themselves, our main goal is to protect the healthy ones you have left by eating right and managing your blood pressure so your kidneys can keep doing their job for a very long time. |
